# Study on Microstructures and Properties of FeCoNiCuAlSi_x_ High-Entropy Alloy Composite Coatings by Laser Cladding

**DOI:** 10.3390/mi16111211

**Published:** 2025-10-24

**Authors:** Xinyu Zhang, Chun Guo, Guangcan Huang, Zheng Peng, Ruizhang Hu, Qingcheng Lin, Tianyuan Lu

**Affiliations:** 1 College of Intelligent Manufacturing, Anhui Science and Technology University, Chuzhou 239000, China; 18855229559@163.com (X.Z.); guochun@ahstu.edu.cn (C.G.); 17333218178@163.com (Q.L.); 18160816972@163.com (T.L.); 2 Research and Development Center of Flexible Display Materials at Kaisheng Technology Co., Ltd., Bengbu 233000, China; huanggc1027@163.com; 3 College of Chemistry and Materials Engineering, Anhui Science and Technology University, Bengbu 233000, China; hurzh@ahstu.edu.cn

**Keywords:** laser cladding, high-entropy alloy, Si, performance, corrosion resistance

## Abstract

FeCoNiCuAl high-entropy alloys exhibit remarkable mechanical properties; nevertheless, these materials struggle to withstand harsh environments because of their insufficient resistance to wear and corrosion. The addition of Si can significantly enhance the alloy’s high-temperature performance, hardness, and wear resistance, thereby making it more suitable for applications in high-temperature or corrosive environments. To overcome these drawbacks, this research investigates how varying Si content affects the microstructure and properties of FeCoNiCuAl coatings. Composite coatings of FeCoNiCuAlSi_x_ (x = 0, 0.5, 1.0, 1.5, 2.0) were fabricated on 65 Mn substrates using laser cladding. Various testing methods, including metallographic microscopy, Vickers hardness testing, friction and wear testing, and electrochemical analysis, were employed to examine the phase structure, microstructure, and hardness of the coating. It is observed that the FeCoNiCuAl coating begins with a uniform FCC phase structure. However, as the Si content increases, a phase transformation to the BCC structure occurs. The microstructure is primarily composed of isometric crystals and dendrites that become finer and more compact with higher Si content. For the FeCoNiCuAlSi_2.0_ coating, the microhardness reaches 581.05 HV_0.2_. Additionally, wear resistance shows a positive correlation with Si content. Electrochemical testing in NS4 solution shows that the corrosion potential of the coating increases from −0.471 V for FeCoNiCuAl to −0.344 V for FeCoNiCuAlSi_2.0_, while the corrosion current density decreases from 1.566 × 10^−6^ A/cm^2^ to 4.073 × 10^−6^ A/cm^2^. These results indicate that Si addition plays a crucial role in enhancing the mechanical properties and corrosion resistance of FeCoNiCuAl coatings, making them more suitable for high-performance applications in extreme environments.

## 1. Introduction

Laser cladding is a modern surface enhancement technique characterized by its precise energy control, dense coating structure, small heat-affected zone, and high bonding strength when compared to traditional surface modification methods such as electroplating, plasma spraying, and physical vapor deposition. This process involves depositing cladding powder onto the surface of a substrate material in various ways. Upon irradiation with high-energy laser beams, the powder rapidly melts and solidifies in combination with the substrate material, forming a metallurgical bond between the two interfaces [1,2,3,4,5]. Laser cladding technology has the following advantages: (1) It provides high flexibility in the selection of coating materials, allowing it to be adapted to different working environments and requirements; (2) the coating and the substrate can form a good metallurgical bond to provide excellent mechanical properties, high temperature corrosion resistance and other properties; (3) high productivity in practical applications, easy to realize automated production. It has a wide range of applications in the industrial field of material surface modification and coating preparation, and, at the same time, has a very high research and application value, with broad prospects for development [6,7,8,9,10].

To improve the overall performance of laser cladding coatings, the incorporation of reinforcement phases can achieve comprehensive optimization of the coating. Liu et al. [11] explored the influence of WC particles on the surface morphology, microhardness, cracking behavior, and wear/corrosion resistance of Co-based/WC composite coatings. Their results demonstrated that the WC particle content plays a crucial role in crack sensitivity, microhardness, and the wear/corrosion resistance of the coating. Increasing the amount of fine WC particles led to enhanced hardness and wear resistance in the fusion cladding layer, attributed to solid solution strengthening and the formation of finer crystals. Hu et al. [12] employed high-speed laser cladding (HSLC) to produce Ni/WC composite coatings on 304 stainless steel. Their study revealed that the dendritic structure of the coating became finer as WC content increased. The coating mainly consisted of Ni_3_Fe along with reinforcing phases like W_2_C and Cr_23_C_6_. The decomposition of WC particles resulted in fine-grain strengthening and solid solution strengthening, improving the hardness and wear resistance. He et al. [13] investigated the impact of copper mass fractions on the microstructure, phase composition, and corrosion resistance of copper-cobalt alloy composite coatings. They found that 1% copper content led to the densest microstructure and the highest corrosion resistance, while higher copper contents resulted in larger grain sizes and increased porosity. Despite the positive effects of copper, the coating’s properties decreased beyond 1% copper.

High-entropy alloys (HEAs), which are composed of several elements in nearly equal proportions, have gained considerable interest due to their exceptional mechanical and thermal properties. The high configurational entropy of HEAs promotes the formation of solid solutions, which, in turn, contribute to improvements in strength, wear resistance, and corrosion resistance [14,15,16,17,18,19]. For instance, Li et al. [20] investigated the role of Mn in the mechanical properties of AlCrFeNiMnx coatings. They found that Mn significantly reduced the grain size, resulting in enhanced hardness and wear resistance of the coatings. These coatings demonstrated an optimal balance of mechanical properties, with a Vickers hardness of 625 HV, a friction coefficient of 0.42, and low mass loss. In another study, Liu et al. [21] examined the effect of Si content on AlCoCrFeNi HEA coatings, showing that the microhardness increased linearly with Si content. The Si_0.5_ coating reached the highest average microhardness of 8.19 GPa. The primary strengthening mechanisms in these coatings included dislocation strengthening, solid solution strengthening, and grain refinement. Shirali et al. [22] explored the phase evolution, mechanical properties, and corrosion behavior of FeCoNiCu-based high-entropy alloys and their Mo, Si, Cr, and Nb-substituted variants. They found that the FeCoNiCuNb alloy exhibited brittle fracture behavior, with a fracture strength of 458 MPa, indicating some resistance to brittle fracture, but still significantly weaker than ductile alloys. In comparison, FeCoNiCuSi exhibited remarkably high hardness, reaching 863 HV, while FeCoNiCu showed the lowest hardness at 180 HV.

When Si is added as an alloying element into the matrix, it alters the lattice structure of the matrix, resulting in increased lattice distortion, which in turn enhances the solid solution strengthening effect. As the Si content increases, the dislocation density rises, hindering the movement of dislocations, thereby improving the material’s strength. Research on the effects of non-metallic elements Si in high-entropy alloys has been limited. Based on metallurgical principles, non-metallic elements are recognized to significantly influence the structure and properties of high-entropy alloys [23,24,25,26]. This study explores the effect of different Si concentrations in FeCoNiCuAl high-entropy alloy powder, with the goal of optimizing the Si content within the hybrid powder system. The aim is to produce high-entropy alloy composite coatings on a 65 Mn substrate and analyze the microstructure and properties of the resulting coatings.

## 2. Materials and Methods

### 2.1. Experimental Materials

**Figure 1:**
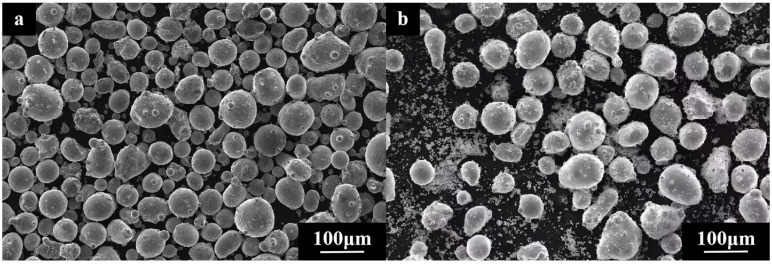
Powder morphology: (**a**) FeCoNiCuAl high-entropy alloy powder morphology; (**b**) mixed morphology of FeCoNiCuAl high-entropy alloy powder and Si powder.

The test substrate chosen was a Φ60 mm × 15 mm 65 Mn specimen. Prior to testing, the substrate surface was cleaned using 240 mesh and 600 mesh sandpaper to eliminate the oxide layer and oil, followed by rinsing with anhydrous ethanol. For the test, FeCoNiCuAl high-entropy alloy powder (particle size: 45–105 μm) and Si powder (purity > 99.99%, 300 mesh) were selected. According to the test program, different mass fractions of the powders were weighed using an electronic balance for the preparation. The test powder composition is shown in Table 1. The powders in different ratios were placed in a ball mill for ball milling and mixing. The ball-to-powder ratio was 3:1, and zirconia balls were used as the milling media. The ball mill was operated at a speed of 150 r/min for 2 h in an open environment. After milling, the mixed powders were dried for 1 h before testing. Figure 1 shows the morphology of the test powder under the scanning electron microscope (Zeiss EVO18 produced by Zeiss, a German optical company based in OberCohen, Baden-Württemberg, Germany), it can be seen that the powder composition is uniform with a small amount of agglomeration phenomenon.

The test involves pre-setting five different types of powder onto the surface of the substrate to form a 1.5 mm powder bed. The laser cladding equipment consists of an IPG 6000 W laser and a KUKA robot. By controlling the process through parameter mapping, the optimal settings were determined as follows: a laser power of 1500 W, scanning speed of 5 mm/s, 1.5 mm overlap, 40 mm focal length, 5 mm spot diameter, and an argon shielding gas flow rate of 15 L/min.

### 2.2. Test Methods and Characterization

Samples with dimensions of 10 mm × 5 mm × 5 mm, 20 mm × 20 mm × 8 mm, and Φ25 mm × 15 mm were fabricated through the EDM wire cutting process after laser cladding. Microhardness measurements were performed with an HV1000Z tester, with readings taken at 0.15 mm intervals along the cross-section from the coating surface to the substrate. The distance from each measurement point to the coating surface was also recorded. Metallographic analysis was performed by etching with aqua regia (HCl:HNO_3_ = 3:1), followed by rinsing with anhydrous ethanol before examining the samples under a microscope. X-ray diffraction was carried out using a Cu target X-ray tube with a step size of 0.02 degrees, a scanning speed of 4°/min, and a scan range from 10° to 90°. The diffraction data were analyzed using Jade 6 software. For friction and wear tests, a friction tester with a GCr_15_ steel friction vice was used. The tests were conducted at a radius of 6 mm, with a rotational speed of 500 r/min, a load of 40 N, and a duration of 30 min. The sample mass was measured before and after the wear tests with an electronic balance with 0.1 mg precision, and the tests were repeated three times for consistency. Wear patterns on the samples were observed using a Zeiss EVO18 scanning electron microscope. Electrochemical tests were performed using a CHI 660E produced by Shanghai Chenhua, Shanghai, China, electrochemical workstation with NS4 solution as the electrolyte, conducting Tafel curve and electrochemical impedance spectroscopy tests.

## 3. Results and Discussion

### 3.1. Morphology of Laser-Clad HEA Specimens

**Figure 2:**
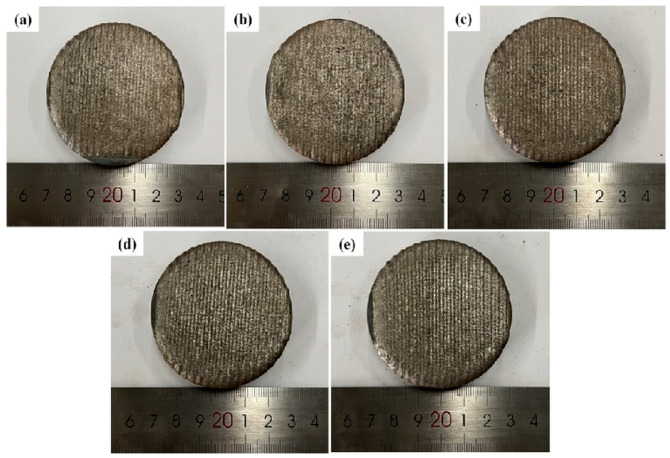
Macroscopic morphology of FeCoNiCuAlSi_x_ high-entropy alloy composite coating: (**a**) FeCoNiCuAl; (**b**) FeCoNiCuAlSi_0.5_; (**c**) FeCoNiCuAlSi_1.0_; (**d**) FeCoNiCuAlSi_1.5_; (**e**) FeCoNiCuAlSi_2.0_.

**Figure 3:**
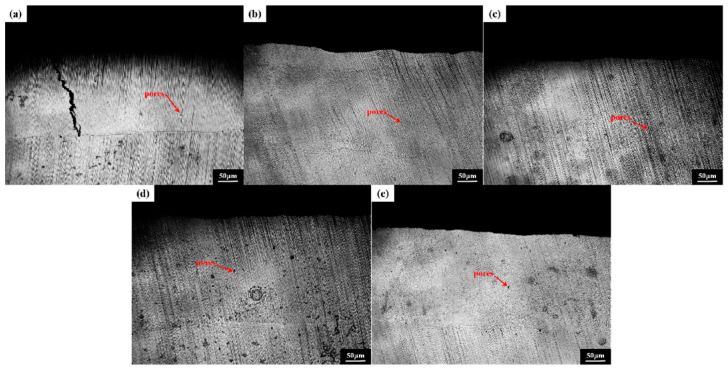
Cross-sectional morphology of FeCoNiCuAlSi_x_ high-entropy alloy composite coating: (**a**) FeCoNiCuAl; (**b**) FeCoNiCuAlSi_0.5_; (**c**) FeCoNiCuAlSi_1.0_; (**d**) FeCoNiCuAlSi_1.5_; (**e**) FeCoNiCuAlSi_2.0_.

Figure 2 illustrates the overall surface structure of the multi-pass FeCoNiCuAlSi_x_ high-entropy alloy composite coating, while Figure 3 displays its cross-sectional structure. As the Si content increases, the coating surface becomes smoother, more uniform, and exhibits improved formability. This enhancement can be attributed to Si, which promotes better wettability between the fusion cladding layer and the substrate. By extension, Si is also expected to enhance the wettability between adjacent coating layers, thereby contributing to improved overall surface quality [27].

### 3.2. Physical Phase Analysis of Coatings

**Figure 4:**
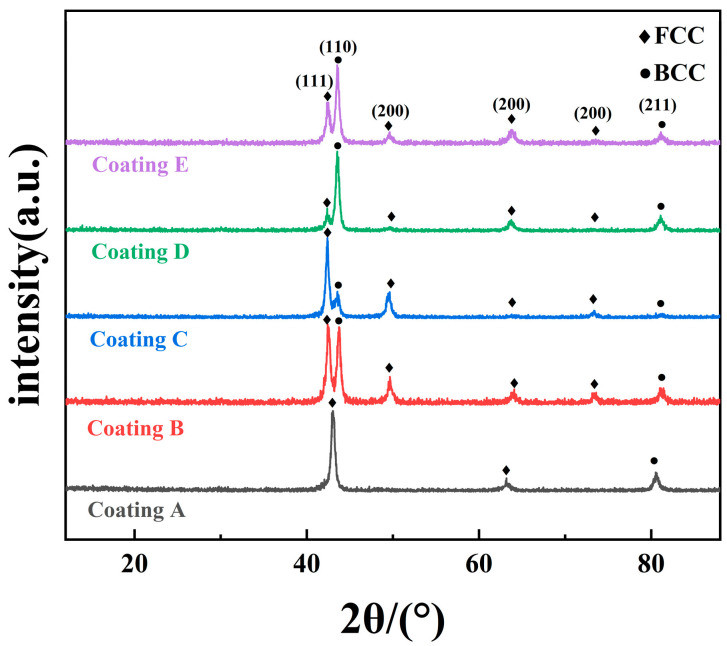
XRD patterns of FeCoNiCuAlSi_x_ high-entropy alloy composite coatings: (Coating A) FeCoNiCuAl; (Coating B) FeCoNiCuAlSi_0.5_; (Coating C) FeCoNiCuAlSi_1.0_; (Coating D) FeCoNiCuAlSi_1.5_; (Coating E) FeCoNiCuAlSi_2.0_.

Figure 4 displays the XRD diffraction pattern of the FeCoNiCuAlSi_x_ high-entropy alloy composite coating, revealing the presence of both BCC and FCC phases. The BCC solid solution phase, being unstable in this alloy system, may undergo phase separation during solidification, resulting in amplitude-modulated decomposition. Under the influence of high temperature and high-entropy, Fe, Ni, and Al atoms aggregate to form a supersaturated BCC solid solution. This initially formed superlattice solid solution undergoes amplitude-modulation decomposition as the solidification temperature and entropy decrease, resulting in a two-phase mixed structure. The diffraction peaks either overlap or separate depending on the relative content of the different solid solutions. The BCC phase solid solution is primarily dominated by one type of solid solution, with the other type present in lower amounts. The FCC phase is a solid solution structure composed of Co, Cr, Fe and Ni. Because of the presence of multiple primary elements, the overall mixing entropy of the alloy increases so that the compatibility between the primary elements increases with it. Since intermetallics are ordered phases with continuous chemical forms and specific crystal structures, high mixing entropy minimizes the generation of multiple complex intermetallics due to phase separation [28]. From Figure 4, it can be observed that the variations in Si content caused a large change in the phase structure, and it can be seen from the diffraction peaks that the diffraction intensity of FCC gradually decreases with the increase in the Si content, and Si helps to improve the dot vacancies during the crystallization of the alloys and reduces the lattice distortion, which suggests that Si is an element that promotes the formation of BCCs [29].

### 3.3. Microstructural Analysis of Coating

**Figure 5:**
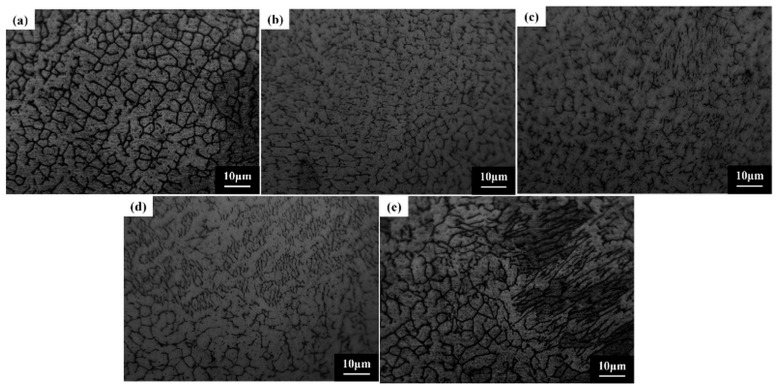
Microstructure and morphology of FeCoNiCuAlSi_x_ high-entropy alloy composite coatings: (**a**) FeCoNiCuAl; (**b**) FeCoNiCuAlSi_0.5_; (**c**) FeCoNiCuAlSi_1.0_; (**d**) FeCoNiCuAlSi_1.5_; (**e**) FeCoNiCuAlSi_2.0_.

Figure 5 depicts the microstructural characteristics of the FeCoNiCuAlSi_x_ high-entropy alloy composite coating. The variation in silicon content plays a crucial role in influencing microstructure evolution by affecting nucleation and growth behavior during the laser cladding and solidification process. In the case of the FeCoNiCuAl coating, the microstructure consists primarily of equiaxial crystals with large grain sizes and a compact arrangement. The high-entropy effect of the alloy facilitates a hysteresis diffusion effect, providing time for grain growth during solidification. This results in a relatively stable and well-defined coarse microstructure, with a uniform distribution of elements and minimal segregation. When silicon is added at 0.5 at%, the microstructure transitions to dendritic crystal growth, with Si-rich regions forming within the dendrites. This segregation occurs due to the larger atomic radius of Si, which induces a local exclusion effect on other elements during cooling, leading to a more controlled solidification process [30]. As the Si content increases to 1.0 at% and 1.5 at%, the dendritic structure becomes more pronounced, with finer, more intricate networks of dendrites forming due to the reduced mobility of the molten alloy. The increase in Si concentration slows the nucleation and growth rates, resulting in denser and more uneven dendritic arms, enhancing the overall structural complexity [31]. XRD analysis confirms the retention of the FCC phase structure, though additional peaks corresponding to silicon-rich phases appear. At the highest Si concentration of 2.0 at%, the microstructure becomes much finer due to an increased nucleation rate, which suppresses individual grain growth and results in a uniform, dense dendritic network. This fine-grained structure significantly enhances properties such as hardness and wear resistance. The solidification mechanism during laser melting and cladding is influenced by the heat transfer direction, causing nucleation to predominantly occur along the cooling front [32]. Silicon addition increases the nucleation rate and leads to more complex phase formations. with Si-rich phases interspersed within the dendritic matrix. With the finer dendritic networks becoming more pronounced as the Si content increases, improving the overall performance of the coating.

### 3.4. Microhardness of Coatings

**Figure 6:**
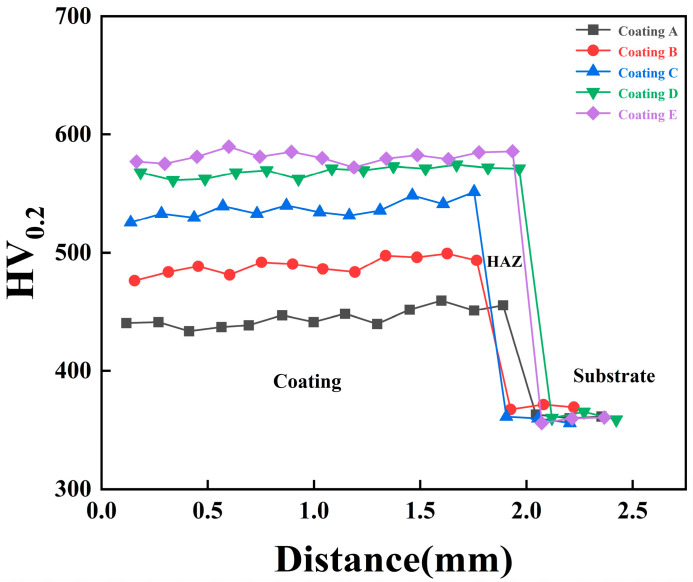
Microhardness of FeCoNiCuAlSi_x_ high-entropy alloy composite coatings: (Coating A) FeCoNiCuAl; (Coating B) FeCoNiCuAlSi_0.5_; (Coating C) FeCoNiCuAlSi_1.0_; (Coating D) FeCoNiCuAlSi_1.5_; (Coating E) FeCoNiCuAlSi_2.0_.

Figure 6 illustrates the microhardness variation in the FeCoNiCuAlSi_x_ high-entropy alloy composite coating. The data demonstrate a clear positive correlation between Si content and microhardness, reaching a peak at x = 2.0. At this composition, the average microhardness attains 581.05 HV_0.2_. The smaller atomic radius of Si compared to other constituent elements leads to an increase in crystal lattice distortion within the coating. This distortion, caused by atomic size mismatch, results in the accumulation of lattice defects. As Si, with its smaller atomic radius, occupies interstitial sites within the lattice, it significantly enhances the lattice density and uniformity. However, this also reduces the coating’s ability to undergo plastic deformation, thereby increasing its microhardness [33]. The microstructure of the alloy coating plays a critical role in determining its hardness. With increasing Si content, the fraction of the BCC phase in the coating grows, contributing significantly to improved hardness. When Si is not present, the coating shows a comparatively low hardness of 445.02 HV_0.2_, mainly because the microstructure is dominated by the softer FCC phase, with only a small amount of the harder BCC phase in the FeCoNiCuAl alloy system. Aluminum is the primary stabilizer of the BCC structure, and the introduction of Si helps reduce Al loss during processing. Moreover, Si is a typical self-melting element that tends to undergo self-oxidation in the molten state. This reaction reduces the overall oxygen content and slows the oxidation of Al, thereby retaining more Al in the alloy and promoting the formation of the BCC phase [34]. Additionally, the increased formation of BCC phases within the FCC matrix results in a pronounced precipitation strengthening effect, which markedly improves the hardness of Si-containing alloy coatings [35]. In conclusion, introducing Si into the alloy coatings increases the proportion of the BCC phase while also inducing significant lattice distortion, both of which contribute positively to strengthening the coatings.

### 3.5. Frictional Wear Behavior of Coatings

#### 3.5.1. Average Coefficient of Friction and Wear Rate

**Figure 7:**
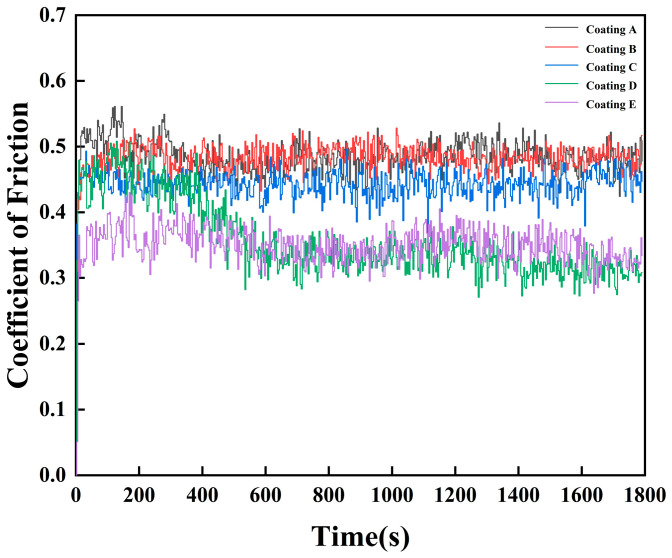
Friction coefficient of FeCoNiCuAlSi_x_ high-entropy alloy composite coating: (Coating A) FeCoNiCuAl; (Coating B) FeCoNiCuAlSi_0.5_; (Coating C) FeCoNiCuAlSi_1.0_; (Coating D) FeCoNiCuAlSi_1.5_; (Coating E) FeCoNiCuAlSi_2.0_.

**Figure 8:**
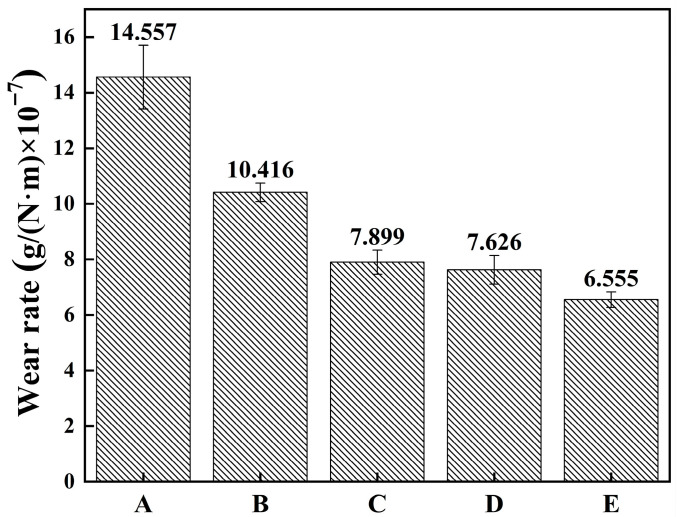
Wear rate of FeCoNiCuAlSi_x_ high-entropy alloy composite coating: (A) FeCoNiCuAl; (B) FeCoNiCuAlSi_0.5_; (C) FeCoNiCuAlSi_1.0_; (D) FeCoNiCuAlSi_1.5_; (E) FeCoNiCuAlSi_2.0_.

Table 2 summarizes the friction and wear performance of FeCoNiCuAlSi_x_ high-entropy alloy composite coatings, while Figure 7 shows the variation in friction coefficient, and Figure 8 presents the corresponding wear rates. The wear behavior can be categorized into two distinct phases: the initial and the steady-state wear stages. In the early phase, friction is unstable due to the uneven interaction between the coating and its counterpart, leading to a sharp increase in the friction coefficient. As the test continues, the system transitions into a more stable wear stage with a relatively constant friction coefficient. During the initial stage, contact occurs mainly at surface asperities, and the fluctuations in friction are largely caused by the gradual removal of these micro-scale protrusions. For the FeCoNiCuAl and FeCoNiCuAlSi_0.5_ coatings, which have lower hardness, plastic deformation of the surface micro-protrusions dominates the wear process, leading to a linear relationship between the friction coefficient and wear time, quickly transitioning into the stable wear stage. However, for coatings with higher hardness, such as FeCoNiCuAlSi_1.0_, FeCoNiCuAlSi_1.5_, and FeCoNiCuAlSi_2.0_, wear is primarily governed by brittle fracture of the micro-protrusions, causing the friction coefficient to deviate from a linear relationship with wear time. High-entropy alloys with predominantly FCC phases typically exhibit lower hardness and higher plasticity. However, an increase in the BCC phase content significantly enhances the hardness and strength of these alloys. The addition of Si refines and densifies the microstructure, which plays a key role in improving the wear resistance of the coatings. Furthermore, as brittle fracture occurs, the contact area of the micro-protrusions with the friction counterpart increases, extending the duration of micro-protrusion contact wear. During the stable wear phase, the friction coefficient decreases continuously as the Si content rises. The average friction coefficients for the FeCoNiCuAl, FeCoNiCuAlSi_0.5_, FeCoNiCuAlSi_1.0_, FeCoNiCuAlSi_1.5_, and FeCoNiCuAlSi_2.0_ coatings were approximately 0.485, 0.481, 0.442, 0.357, and 0.349, respectively, as predicted by the Archard Equation [36], the wear quality of the alloy coatings is inversely related to the microhardness:

(1) $$Q=\frac{KWL}{H}.$$

In the public notice (1), K is the coefficient of friction, W is the applied load, L is the sliding distance, and H is the microhardness.

The combined coating microhardness can be concluded that Si content is positively correlated with the wear resistance and microhardness of the coating. This behavior is consistent with the Hall-Petch relationship, which suggests that the hardness of a material increases with a decrease in the average grain size. In high-entropy alloys with increased Si content, the microstructure becomes finer and denser, which enhances hardness and strength. As the Si content rises, the grain boundaries become more resistant to dislocation motion, increasing the material’s hardness and improving wear resistance. Furthermore, the presence of Si particulates contributes to the wear resistance by providing a mechanism for load-bearing during the wear process. As Si particulates act as reinforcing agents, they increase the load-bearing capacity of the coating. The Orowan mechanism, which describes the process of dislocation bypassing by reinforcing particles, plays a key role here [37]. When the dislocations encounter Si particles, they cannot move through them directly, which results in the dislocations bowing around the particles and creating additional strengthening. This phenomenon enhances the coating’s ability to withstand mechanical stresses and improves its wear resistance.

#### 3.5.2. Coating Surface Wear Morphology and Mechanism

**Figure 9:**
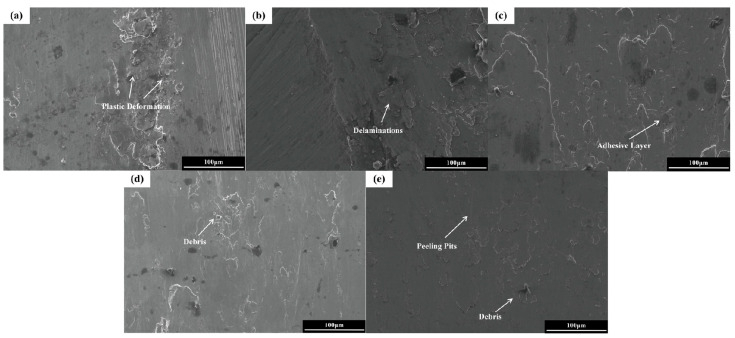
Surface wear profile of specimens with different Si contents: (**a**) FeCoNiCuAl; (**b**) FeCoNiCuAlSi_0.5_; (**c**) FeCoNiCuAlSi_1.0_; (**d**) FeCoNiCuAlSi_1.5_; (**e**) FeCoNiCuAlSi_2.0_.

Figure 9 illustrates the wear scar morphology of FeCoNiCuAlSi_x_ high-entropy alloy composite coatings. In Figure 9a, the wear surface of FeCoNiCuAl displays prominent grooves, accompanied by debonding pits and adhesive layers along the edges of these grooves. These features indicate considerable plastic deformation of the matrix during wear, highlighting distinct signs of adhesive wear [38]. As the Si content increases, the wear on the coating significantly decreases, and the wear surface becomes progressively smoother. For FeCoNiCuAlSi_0.5_ and FeCoNiCuAlSi_1.0_ coatings, a large amount of abrasive debris is observed, and the grooves formed are shallower and wider compared to coatings without Si. The clear delamination at the edges of the grooves in low-Si coatings indicates periodic delamination and fracture during wear. In addition to plastic deformation, small amounts of material flaking occur, and friction, along with solid-phase welding, causes these flakes to form a bonding layer. The wear mechanisms of the FeCoNiCuAl, FeCoNiCuAlSi_0.5_, and FeCoNiCuAlSi_1.0_ coatings are primarily adhesive wear, abrasive wear, and delamination wear. As Si content increases further, the plastic deformation produced by friction decreases, and the depth of the grooves reduces. In the case of the FeCoNiCuAlSi_1.5_ coating, the wear surface exhibits shallow grooves with abundant abrasive debris and adhesive layers, reflecting a significant enhancement in hardness. In Figure 9e, the FeCoNiCuAlSi_2.0_ coating shows fine abrasive grooves along the sliding direction, with more adherent abrasive particles at the edges, indicating that the wear mechanism is mainly abrasive and adhesive. Due to the high hardness of this coating, numerous hard micro-protrusions fracture during wear, acting as tiny abrasives that generate fine grooves on the wear surface.

### 3.6. Corrosion Behavior of Coatings

#### Electrochemical

**Figure 10:**
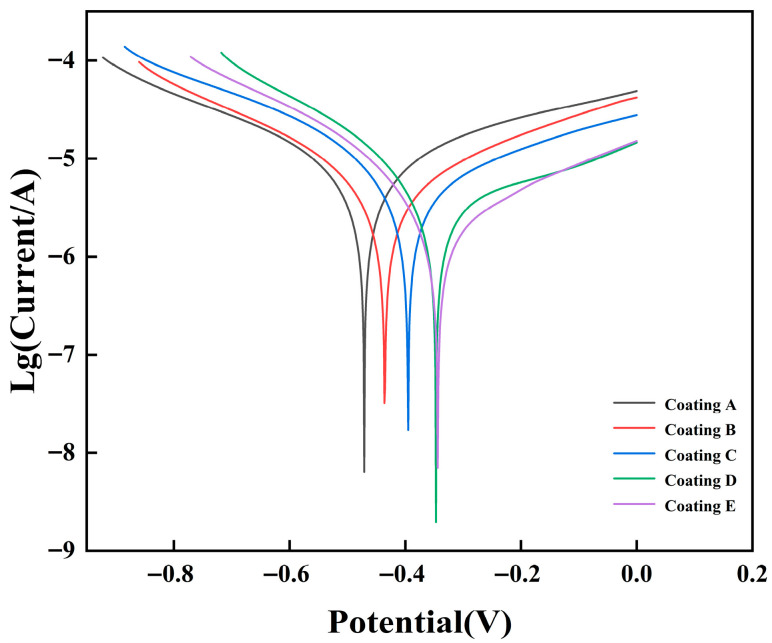
Potentiodynamic polarization curves of the FeCoNiCuAlSi_x_ high-entropy alloy composite coating in NS4 solution: (Coating A) FeCoNiCuAl; (Coating B) FeCoNiCuAlSi_0.5_; (Coating C) FeCoNiCuAlSi_1.0_; (Coating D) FeCoNiCuAlSi_1.5_; (Coating E) FeCoNiCuAlSi_2.0_.

**Figure 11:**
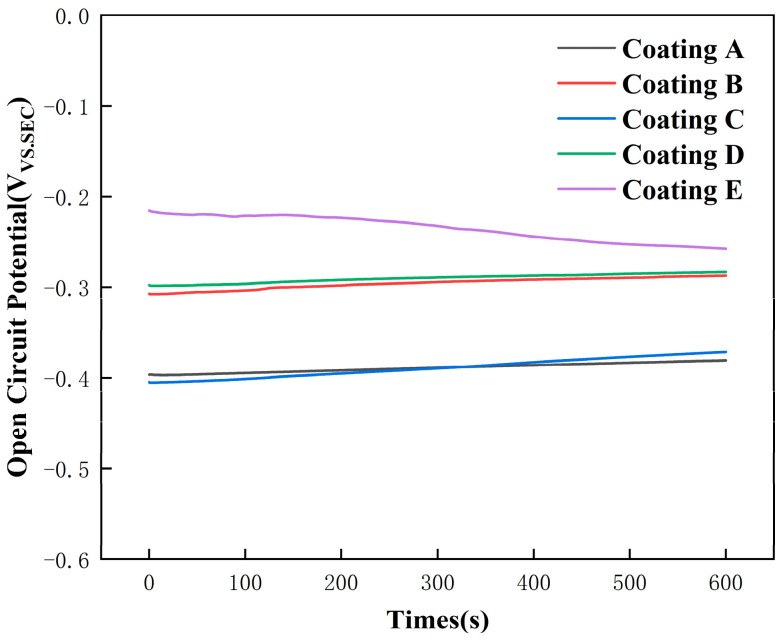
Open circuit potential of FeCoNiCuAlSi_x_ high-entropy alloy composite coating: (Coating A) FeCoNiCuAl; (Coating B) FeCoNiCuAlSi_0.5_; (Coating C) FeCoNiCuAlSi_1.0_; (Coating D) FeCoNiCuAlSi_1.5_; (Coating E) FeCoNiCuAlSi_2.0_.

Figure 10 displays the potentiodynamic polarization curves for FeCoNiCuAlSi_x_ high-entropy alloy composite coatings in an NS4 solution. The reason for choosing NS4 solution in this study is that it simulates the chemical composition of soil pore water, making it widely applicable to corrosion behavior in practical use environments. Figure 11 shows the open circuit potential of the FeCoNiCuAlSi_x_ high-entropy alloy composite coating. A saturated calomel electrode (SCE) with a Lujin capillary tube is used as the reference electrode, the test sample is used as the working electrode, and a platinum plate is used as the auxiliary electrode. The electrochemical corrosion parameters, obtained through the Tafel extrapolation method [39], are presented in Table 3. These parameters, based on electrochemical corrosion theory, offer valuable insights into the coatings’ corrosion resistance [40], the corrosion potential (Ecorr) of a material indicates its susceptibility to corrosion, with higher values correlating to a lower tendency for corrosion and dissolution in a corrosive environment. The corrosion current density (Icorr) reflects the actual corrosion rate, with a larger Icorr indicating a faster corrosion rate and lower corrosion resistance, and a smaller Icorr signifying better resistance. Table 3 shows that the composite coatings with added Si exhibit a higher corrosion potential than the FeCoNiCuAl high-entropy alloy coating. Additionally, as Si content increases, the corrosion current density decreases, dropping by an order of magnitude compared to the Si-free coatings. The improved corrosion resistance is primarily attributed to the formation of a dense Al_2_O_3_ passivation layer on the FeCoNiCuAl alloy, which enhances its protective properties. Furthermore, Si plays a crucial role during the solidification and microstructural evolution of the coating. Si acts as a heterogeneous nucleant during solidification and is subsequently pushed toward the grain boundaries due to surface tension effects. These segregated Si particulates pin the grain boundaries through the zener pinning mechanism, which effectively inhibits grain boundary migration. Grain boundaries typically possess higher surface energy compared to grains and sub-grains, making them more susceptible to corrosive attack. However, the presence of Si particulates along the grain boundaries reduces the boundary energy and provides additional resistance to localized corrosion at these high-energy sites. This microstructural stabilization, combined with the formation of a dense and stable passivation film [41], results in significantly improved corrosion resistance of the coatings in NS4 solution. Based on the data in Figure 10, it can be concluded that Si incorporation significantly boosts the coating’s corrosion resistance.

## 4. Conclusions

(1) The FeCoNiCuAlSi_x_ high-entropy alloy composite coating exhibits a dual-phase structure with both BCC and FCC phases. The incorporation of Si significantly alters the phase structure, promoting the formation of the BCC phase. Additionally, Si plays a key role in reducing lattice defects and improving point vacancies during the crystallization process, thereby enhancing the overall structural integrity.

(2) The microstructure of the FeCoNiCuAlSi_x_ alloy coatings predominantly consists of isometric crystals or dendritic formations. As Si content increases, the nucleation rate within the alloy is enhanced. Si acts as a core for nucleation, slowing down the melt’s fluidity, increasing the nucleation rate, and resulting in slower crystal growth. This leads to a denser and more uniform microstructure, particularly observed in the FeCoNiCuAlSi_2.0_ coating.

(3) There is a direct correlation between the microhardness of the FeCoNiCuAlSi_x_ alloy composite coatings and the Si content. The addition of Si promotes the transformation of the FCC phase to the BCC phase, causing precipitation strengthening. This transformation increases the proportion of the BCC phase, induces significant lattice distortion, and results in a notable enhancement of the hardness of the Si-containing coatings. The hardness of the FeCoNiCuAlSi_x_ (x = 0, 0.5, 1.0, 1.5, 2.0) coatings increased by 22.99%, 32.56%, 50.06%, 58.98%, and 61.90%, respectively, compared to the substrate.

(4) Increasing the Si content enhances the wear resistance of FeCoNiCuAlSi_x_ high-entropy alloy composite coatings. While FCC-phase-dominated alloys generally exhibit lower hardness and greater plasticity, the incorporation of Si promotes the formation of BCC phases. This phase transformation, combined with Si-induced microstructural refinement, leads to increased hardness and improved resistance to wear. The wear rate of FeCoNiCuAlSi_2.0_ coating decreased by 55% compared to FeCoNiCuAlSi coating.

(5) The corrosion behavior of FeCoNiCuAlSi_x_ coatings in NS4 solution improves progressively with higher Si content. An increase in corrosion potential and a marked decrease in corrosion current density are observed. The FeCoNiCuAlSi_2.0_ coating shows an order-of-magnitude reduction in current density compared to the Si-free counterpart, highlighting the critical role of Si in enhancing the alloy’s corrosion resistance.

## Figures and Tables

**Table 1:** Chemical composition of FeCoNiCuAl high-entropy alloy with different Si contents/wt%.

	Si	Fe	Cu	Ni	Al	Co
0 Si	0	21.42	24.12	22.47	10.49	Bal.
0.5% Si	0.5	21.42	24.12	22.47	10.49	Bal.
1% Si	1	21.42	24.12	22.47	10.49	Bal.
1.5% Si	1.5	21.42	24.12	22.47	10.49	Bal.
2% Si	2	21.42	24.12	22.47	10.49	Bal.

**Table 2:** FeCoNiCuAlSi_x_ high-entropy alloy composite coating friction and wear data.

Coating	Average Coefficient of Friction	Amount of Wear/g	Wear Rate g/(N·m)
FeCoNiCuAl	0.485	0.0354	1.566 × 10^−6^
FeCoNiCuAlSi_0.5_	0.481	0.0239	1.057 × 10^−6^
FeCoNiCuAlSi_1.0_	0.442	0.0171	7.564 × 10^−7^
FeCoNiCuAlSi_1.5_	0.357	0.0166	7.342 × 10^−7^
FeCoNiCuAlSi_2.0_	0.349	0.0142	6.281 × 10^−7^

**Table 3:** Electrochemical corrosion parameters of the FeCoNiCuAlSi_x_ high-entropy alloy composite coating in NS4.

Coatings	Ecorr/V	Icorr/A/cm^2^
FeCoNiCuAl	−0.471	1.039 × 10^−5^
FeCoNiCuAlSi_0.5_	−0.436	6.442 × 10^−6^
FeCoNiCuAlSi_1.0_	−0.395	4.823 × 10^−6^
FeCoNiCuAlSi_1.5_	−0.347	4.169 × 10^−6^
FeCoNiCuAlSi_2.0_	−0.344	4.073 × 10^−6^

## Data Availability

The corresponding author declares on behalf of all authors that all raw data and materials discussed in the study will be freely accessible to any researcher who wants to utilise them for non-commercial research without compromising participant privacy. The corresponding author additionally provides information on where if applicable, data might be accessed to support the findings described in the paper.
